# Recurrent Zenker’s Traction Diverticulum After Cervical Spine Surgery: Lessons Learned

**DOI:** 10.7759/cureus.56111

**Published:** 2024-03-13

**Authors:** Sara Yang, Shaley Albaugh, Swati Mehrotra, Eric Thorpe, Steven Charous

**Affiliations:** 1 Otolaryngology, Head and Neck Surgery, Oregon Health & Science University, Portland, USA; 2 Otolaryngology, Head and Neck Surgery, University of Virginia, Charlottesville, USA; 3 Pathology, Hines VA Hospital, Maywood, USA; 4 Otolaryngology, Head and Neck Surgery, Loyola University Medical Center, Maywood, USA

**Keywords:** esophagram, dysphagia, cervical spine surgery, zenker's diverticulum, pharyngeal diverticulum

## Abstract

This is a report of our institutional experience regarding pharyngoesophageal diverticula formation following anterior cervical spine surgery (ACSS).

It is a retrospective chart review of institutional patients from January 2008 to May 2020. Patients at our institution were identified by our two senior authors. Inclusion criteria included patients > 18 years old, a history of prior ACSS, and a confirmed diagnosis of pharyngoesophageal diverticulum with radiographic imaging.

Three patients were identified to have an ACSS-related diverticulum. The case presentations describe surgical management and the subsequent postoperative course. One patient had a particularly complicated course with recurrent diverticulum formation despite prior excision. The patient continued to have dense scar tissue adhering the posterior esophageal wall to the nearby cervical spine plates, despite prior excision and rotation of nearby tissue. This difficult case demonstrated the need for an open and aggressive approach.

ACSS-related diverticula that form in patients with a history of prior anterior cervical spine surgery appear to be a form of traction diverticulum due to dense scar tissue that adheres the pharyngoesophageal mucosa to the adjacent cervical spinal plate. This type of diverticulum differs from Zenker's diverticulum. Surgical management is recommended to resolve patients’ symptoms.

## Introduction

Since the advent of anterior cervical spine surgery (ACSS) as a utilized technique for degenerative disorders and spine trauma in 1958, otolaryngologists have evaluated patients with complaints of dysphagia following this surgery [1]. While overall morbidity rates are low, dysphagia is the second most common complication with an estimated incidence of approximately 54% one month postoperatively and 18.6% at six months [2].

A rare complication causing dysphagia post-ACSS is the development of a pharyngoesophageal diverticulum. Patients typically present with symptoms of progressive dysphagia months or even years following surgery [3]. The ACSS-related diverticula can lead to serious sequelae that include weight loss, abscess formation, pneumonia, mediastinitis, and sepsis [4].

Over the past 12 years, our institution has treated three patients with ACSS-related diverticulum secondary to prior cervical spine surgery. Pre-operative imaging revealed a pharyngeal diverticula near the site of prior cervical fusion that radiologically appeared different from Zenker’s diverticulum. All three of these patients underwent resection of these diverticula via an external approach that confirmed the causal relationship of the ACSS to the development of a diverticulum that was distinctly different from Zenker’s diverticulum. However, one recurred twice despite complete resections. Due to the rare incidence of this complication, we are reporting this in the literature to help further understanding of this process and discuss the issues that confront its treatment.

## Case presentation

Case 1

A 60-year-old woman presented with a history of worsening dysphagia, regurgitation, and difficulty breathing approximately 12 months after a C4-C5 fusion via an anterior approach. Her initial surgery was complicated by the development of right-sided vocal cord paralysis. Video swallow evaluation revealed a diverticulum of the upper esophagus located adjacent to the spinal fusion plates measuring 2.4 x 1.2 cm. The open approach revealed a diverticulum that was scarred down to nearby cervical spinal plates. After the diverticulum was carefully separated, a cricopharyngeal myotomy was performed. The diverticulum was excised using the stapler. To prevent re-adherence, sternocleidomastoid muscles were rotated between the esophagus and plates. No leak or residual diverticulum was noted on her postoperative esophagram study. The patient’s dysphagia was resolved during her postoperative follow-up a month after surgery.

**Figure 1:**
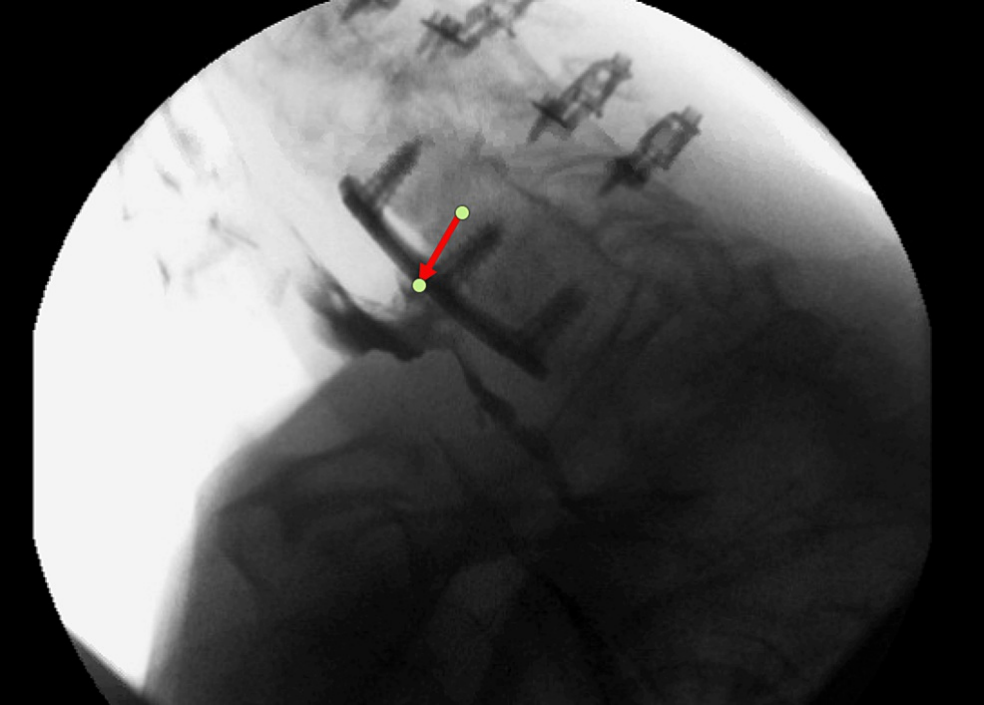
Case 1: recurrent diverticulum imaging Videofluoroscopic swallow study after re-development of symptoms. The red arrow points to the recurrent diverticulum densely adherent to the cervical spine hardware.

She subsequently returned to the clinic a year later with a three-month complaint of dysphagia, choking on phlegm, and recent five-pound weight loss. A videofluoroscopic swallow study confirmed a recurrent diverticulum measuring 7 mm at the site of the previous excision and adherent to the nearby cervical plate (Figure 1).

**Figure 2:**
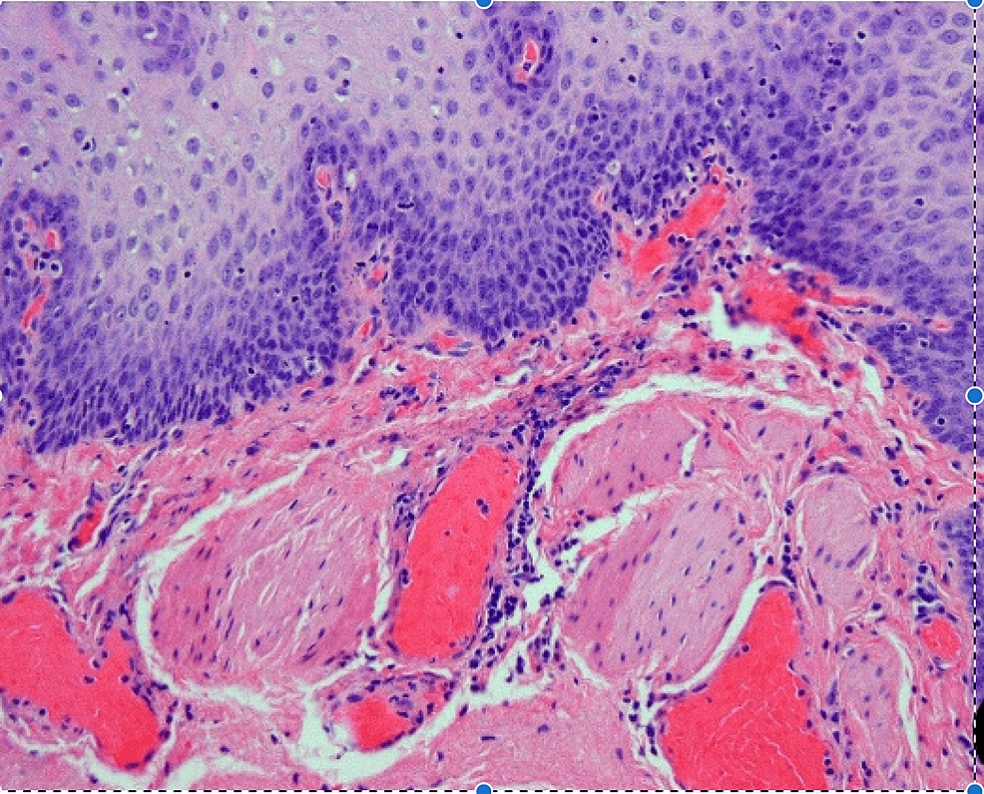
Case 1: histopathology Squamous epithelium-lined diverticulum. Note the absence of muscularis propria in the wall.

The patient was taken back for a transcervical esophageal diverticulum excision. Preoperative coordination with the neurosurgical team was obtained to remove the spinal hardware at the time of resection. Intraoperatively, extensive dense scar tissue encased the posterior aspect of the cervical esophagus, obliterating any planes. The esophageal diverticulum was noted to come off the posterolateral wall of the esophagus and was adherent to the cervical spine. The mucosa of the diverticulum was paper-thin, fragile, and tore easily. Figure 2 demonstrates the histopathology of the ACSS-related diverticulum as a false diverticulum that does not involve the muscular layer.

The diverticulum was separated from the spinal hardware, which had minimal true plate exposure. No perforation secondary to the hardware was noted. The diverticulum was completely resected and the esophageal opening from the resection was primarily closed. Neurosurgery evaluated the patient intraoperatively and deferred cervical plate removal due to poor exposure, dense scar tissue, and lack of obvious tissue planes. Next, an omohyoid muscle flap and sternohyoid muscle flap were rotated into the wound and sutured in place for tissue coverage between the cervical plate and the repaired esophagus. The patient was discharged with a nasogastric tube in place, a drain, and a strict nothing-by-mouth (NPO) status. 

She was admitted postoperatively for a wound infection secondary to an esophageal leak at the site of the previous diverticulum that was treated with IV antibiotic therapy. No residual diverticulum was noted on the esophagram. Wound cultures grew Streptococcus milleri. Her clinical course improved with IV antibiotic therapy and packing of the wound. She subsequently was evaluated by speech pathology with a video swallow study that did not reveal an esophageal leak. The patient was started on oral intake but called a few days afterward, concerned about recurrent infection. She was re-admitted for IV antibiotic therapy. A barium swallow study revealed a contained esophageal leak adjacent to her prior cervical spinal plates. Cultures grew Streptococcus milleri again now with Enterococcus faecalis. A percutaneous endoscopic gastrostomy tube was placed to help the patient maintain strict NPO status. The neck wound was packed with iodoform and the patient was started on long-term IV antibiotic therapy per infectious disease recommendations. The wound was closed by secondary intention successfully and one month later, an esophagram demonstrated no further leak, and oral intake was reinstated.

The patient did well for six months but presented yet again with worsening dysphagia for solids. A barium esophagram demonstrated a recurrence of the diverticulum in the same area. An open approach was deemed to have too high of a potential complication rate and thus she was brought to the OR for an endoscopic approach. A small fistulous tract was noted and a needle knife was used to divide the septum between the fistula tract and the true lumen. A gentle balloon dilation was performed. A small posterior esophageal outpouching without retention of contrast was noted in the postoperative esophagram. Six months postoperatively she denies any dysphagia or regurgitation. She is scheduled for follow-up on an as-needed basis.

Case 2

**Figure 3:**
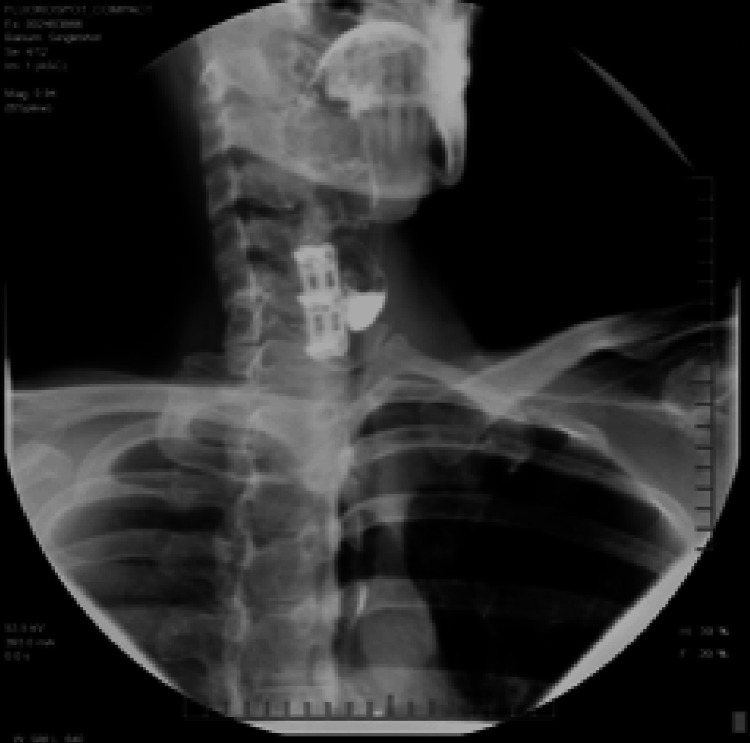
Case 2: imaging Barium esophagram demonstrating a 4.1 x 1.3 cm diverticulum near cervical spine hardware, initially suspected to be a left lateral Killian-Jamieson diverticulum.

A 43-year-old woman presented with worsening dysphagia, choking, regurgitation, and hoarseness that developed about 15 months after anterior cervical fusion. She was diagnosed by a radiologist with a 4.1 x 1.3 cm left lateral Killian-Jamieson diverticulum following a barium swallow study, seen in Figure 3.

**Figure 4:**
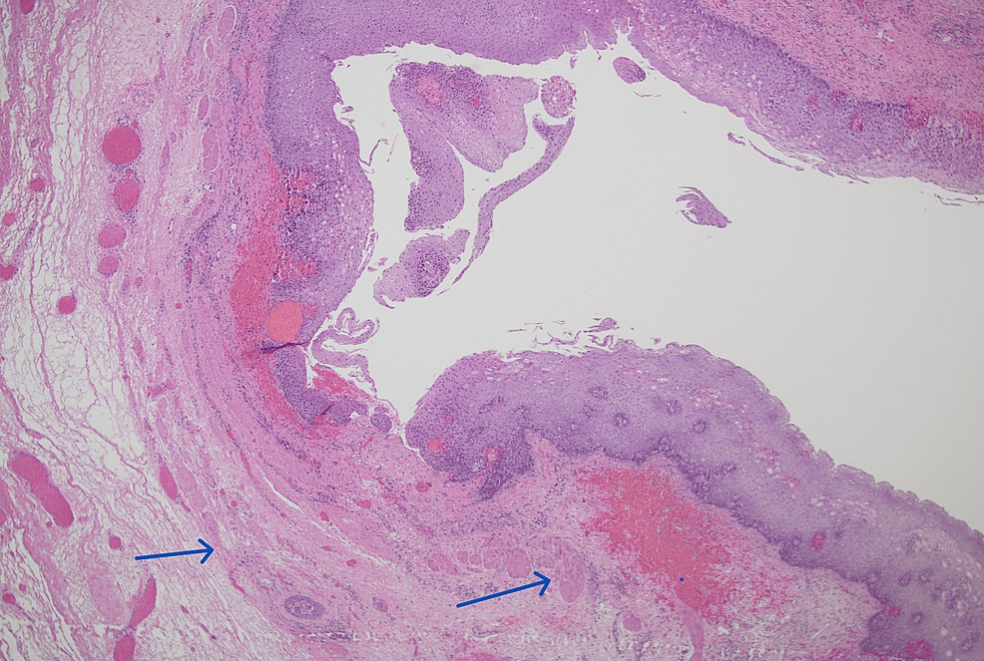
Case 2: histopathology Hypertrophied bundles of muscularis mucosae are evident in the subepithelial region of this ACSS-related diverticulum. Blue arrows show two layers of thickened muscularis mucosae. ACSS: anterior cervical spine surgery

She subsequently underwent an open resection where the pharyngoesophageal diverticulum was identified to be adherent to the cervical spine hardware. Figure 4 shows how this diverticulum was a true diverticulum with the muscular layer present.

The diverticulum was stapled at its neck and the surrounding fascia was re-approximated to separate the esophagus from the spinal plate. A cricopharyngeal myotomy was performed. The patient recovered without complications and is asymptomatic four years post-operatively. 

Case 3

**Figure 5:**
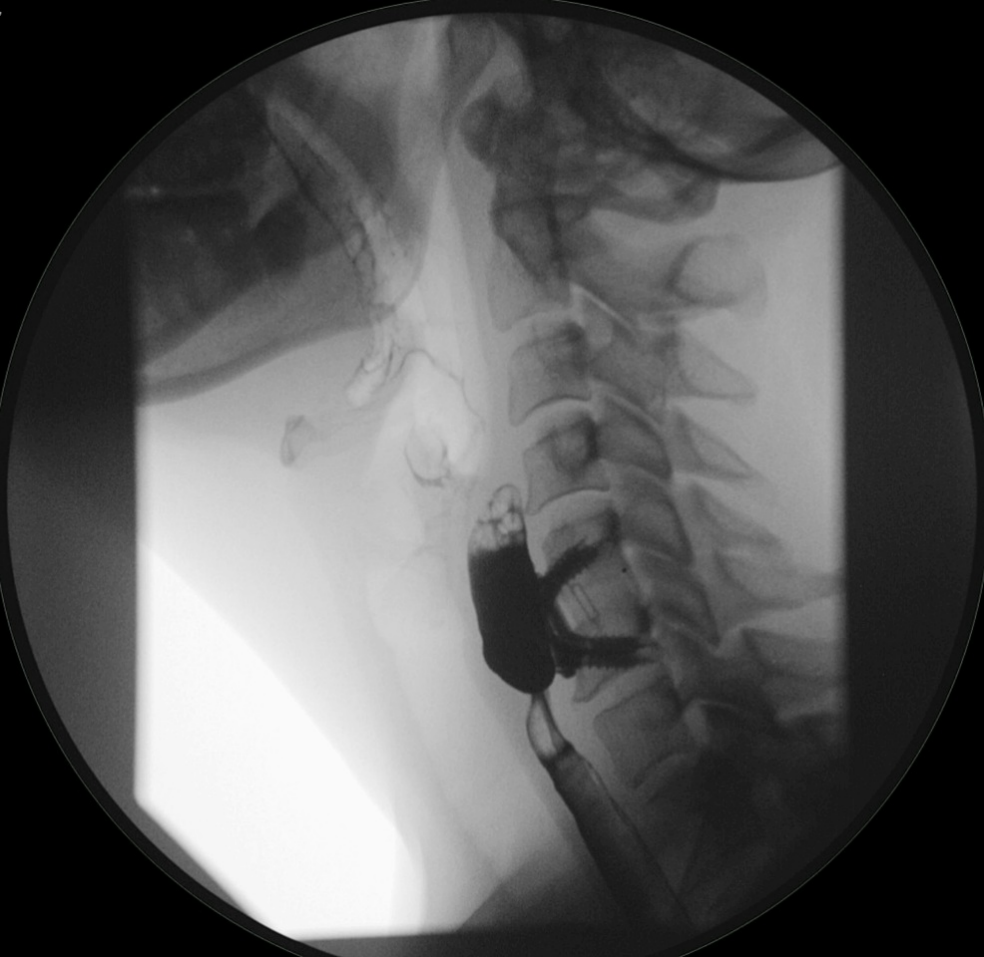
Case 3: imaging Videofluoroscopic swallow study demonstrating a 3.0 x 1.3 x 1.0 cm diverticulum near cervical spine hardware.

A 54-year-old woman presented with a six-month history of worsening dysphagia and globus sensation. She had a prior C5-C6 cervical fusion five years earlier. A videofluoroscopic swallow study revealed a right lateral diverticulum of the hypopharynx measuring 3.0 x 1.3 x 1.0 cm, as seen in Figure 5.

**Figure 6:**
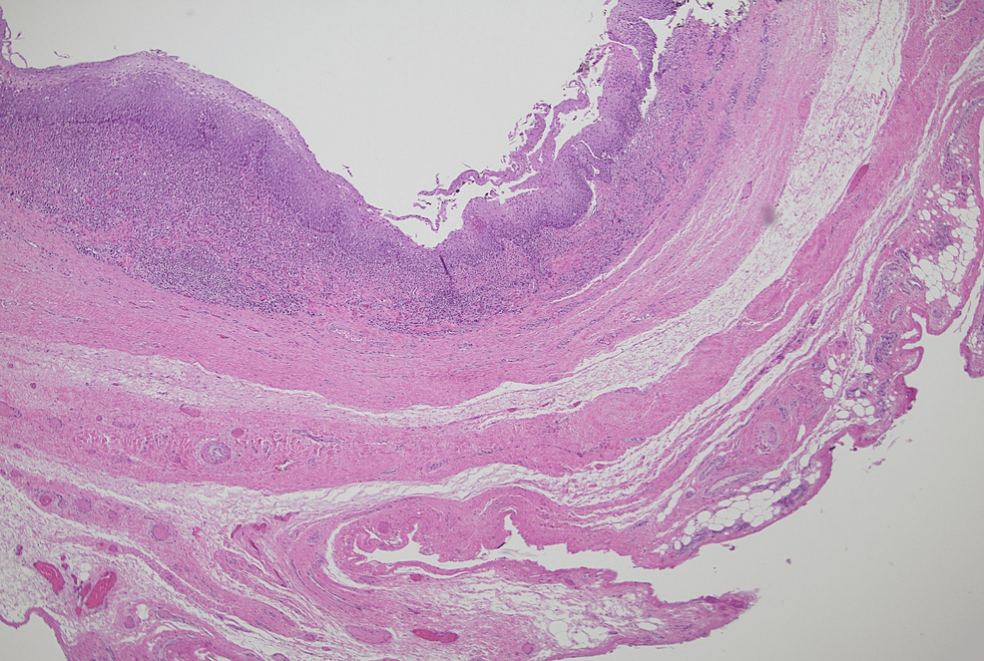
Case 3: histopathology Squamous epithelium-lined diverticulum with dense chronic inflammation in the wall. Muscularis mucosae is not evident. Rather, there are thick collagen bands in the wall.

She underwent an open resection. The hypopharyngeal diverticulum was severely adherent to the spinal plate and dissected free of it. A GI stapler was used to simultaneously ligate and excise the pouch. Figure 6 also demonstrates the presence of the muscular layer in the diverticulum.

A cricopharyngeal myotomy was performed. The patient followed up a month after surgery and reported that her dysphagia was completely resolved.

## Discussion

Esophageal diverticula can be categorized into two types: true and false. True diverticula contain all layers of the esophageal wall, which includes the mucosa, submucosa, muscularis, and adventitia [4-5]. False diverticula only contain the mucosa and submucosa [4]. Zenker’s diverticula are a type of false diverticula occurring in the general population between 0.01% and 0.11% [6-7]. They are classified as pulsion diverticula resulting from inadequate relaxation of the upper esophageal sphincter. Increased intraluminal pressure superior to the contracted CP muscle causes a herniation of mucosa through Killian’s triangle, the weakest point in the cervical esophagus located between the inferior constrictor and CP muscle [4,8-9].

The pathophysiology of an ACSS-related diverticulum is incompletely understood. The hypothesis was that it was generated due to a traction force produced by fibrous scar tissue [10], making it similar to a traction diverticulum. This type of diverticulum usually occurs in the mid-portion of the esophagus near the tracheal bifurcation [5]. It can develop when there is an external force pulling on the esophageal wall, often secondary to infection with tuberculosis or histoplasmosis [4-9]. Traction diverticula are true diverticula involving all layers [4].

Our histological evaluation of the resected ACSS-related diverticula demonstrated diverticula with the muscularis propria present in two cases and absent in one case. This is similar to what has been previously concluded in literature with some authors describing ACSS-related diverticula as a false diverticulum [10-13] versus a true diverticulum [14]. Based on common intraoperative findings of a dense scar band adhering the ACSS-related diverticula to the adjacent spinal plate, it is likely that ACSS-related diverticula are a variant of the pulsion diverticulum. The muscular layer of the pharynx or esophagus may be inadvertently injured during cervical spine surgery retraction, exposing the underlying submucosa. Adhesions form between this submucosa and the cervical hardware because of this trauma followed by inflammation. These adhesions may subsequently pull on the esophagus during swallowing, resulting in an outpouching or diverticulum. Depending on where and how the adhesions pull, the muscular layer may or may not be involved, resulting in a variation of the type of diverticulum formed.

The incidence of ACSS-related diverticula is unknown in the literature. However, dysphagia following ACSS has been previously investigated. Siska et al. found that patients undergoing ACSS had a significant increase in the degree of dysphagia based on Swallowing Quality of Life (SWAL-QOL) scores three weeks after surgery compared to patients undergoing a posterior lumbar approach [15]. A prospective longitudinal study by Mendoza-Lattes et al. found that esophageal retraction may produce a degree of ischemia, and its release may lead to a reperfusion injury that eventually compromises esophageal motility [16]. It was also discovered that patients who reported dysphagia following surgery had significantly higher than average intraluminal pressures in comparison with those who did not present with this symptom [16]. The majority of postoperative dysphagia is temporary and resolves within a few months.

It appears though, that there remains a portion of patients that continue to complain of long-term dysphagia. A retrospective review of 74 patients on average 7.2 years after ACSS by Yue et al. found that 35.1% of patients continue to report some degree of dysphagia [17]. Only four of the 26 patients reporting dysphagia had formal swallow studies, none of which demonstrated a diverticulum [17]. It is difficult to say whether these patients were reporting dysphagia due to an ACSS-related diverticulum because not every patient was imaged. However, there is suspicion that some of these patients had undiagnosed diverticula.

Surgical management is usually indicated but ultimately relies on patients’ symptoms and findings. Four main considerations need to be taken into account when planning and executing the surgical procedure. These considerations are: deciding to use an open or endoscopic approach, the need to remove cervical spine hardware, whether to perform a cricopharyngeal myotomy, and whether a muscular flap is necessary for coverage, Endoscopic and open approaches have been previously discussed in the literature. Certainly, an open approach is indicated in any case in which cervical hardware has perforated the diverticulum. The authors of this paper find that an open approach has certain advantages and is favored compared to an endoscopic approach in all initial cases of these traction diverticula. Transcervical exposure allows for separation of the diverticulum from the spinal hardware where it is tethered to scar tissue, evaluation of the cervical spinal plate, and complete removal of the diverticulum. There is concern that in an endoscopic approach if the scar band tethering the diverticulum to the spinal hardware is not addressed, persistent dysphagia, further erosion, and possible perforation may occur over time. The main disadvantage of the open approach is that the dissection may be more difficult due to altered tissue planes from the previous surgery and thus carries with it a higher risk for recurrent laryngeal nerve injury and esophageal fistula. However, the endoscopic approach has been used successfully before, as reported by Dhar et al., and it has the advantages of a shorter operative time, quicker recovery, and the potential to resume oral intake sooner [18]. The decision to pursue an open versus an endoscopic approach should be tailored to each patient as well as other factors such as body habitus and comorbidities.

Cervical plate removal at the time of the diverticulectomy may not be required if infection, extrusion, or other cervical plate issues are not present. However, consultation with a spinal team preoperatively is prudent to determine whether removal of the cervical hardware is an option. If removal is possible without significant morbidity, we believe is it advantageous to remove that foreign body and decrease the risk of recurrent adhesions along with their potential for instigating the reformation of the diverticula.

Following the diverticulectomy, a sternocleidomastoid (SCM) flap, if available, should be rotated between the primarily repaired esophagus and cervical plate or spine This should reduce the likelihood of ACSS-related diverticula redevelopment since the cervical plate and/or previous area of adhesion is no longer in contact with the esophagus directly. If the plates are not removed, covering them with a flap is necessary. If the plates are removed, one can easily argue that without the foreign body present, further adhesions should not occur and a recurrence of the diverticulum should not develop. There is no literature supporting flap coverage in cases when a cervical plate is not present, but the paucity of data due to the limited number of reported cases precludes any conclusions that can be drawn. The SCM flap is readily available in the surgical field and is easily rotated into the area of concern. It is a thicker and wider flap than the omohyoid and sternothyroid flaps and creates a better barrier than them. Our use of the omohyoid and sternothyroid flaps may have been doomed to failure in our first patient, given the ensuing infection postoperatively. Whether the SCM flap would have fared any better in maintaining a barrier between the esophagus and cervical plates is only conjecture.

It is unclear whether a cricopharyngeal myotomy is necessary [19]. We performed cricopharyngeal myotomies in our patients during our open approach. Even though ACSS-related diverticula are hypothesized to be a type of traction diverticulum, it is unknown whether cricopharyngeal dysfunction is also a contributing factor. A scar that tethers the submucosa superiorly to the cricopharyngeal band of muscle may increase the likelihood of diverticula formation. Given that the cricopharyngeal muscle is exposed during the open diverticulectomy, it may be of benefit to address it at the same time to prevent any future dysfunction and the need for repeat surgery. However, as demonstrated in our first case, a potential disadvantage of performing a cricopharyngeal myotomy is that removing this muscular layer does expose more underlying mucosa that could theoretically be a source of future adhesions and tethering. If the diverticulum or further adhesions do recur requiring further surgery, the absence of the additional muscular layer makes closure of the thin, mucosa-only layer difficult and predisposes to a high likelihood of dehiscence and wound infection as again demonstrated in our first patient. In addition, as always, the division of the cricopharyngeal muscle may predispose patients to laryngopharyngeal reflux symptoms. We, therefore, advocate for a cricopharyngeal myotomy only in those patients in whom a definitive bar is noted on fluoroscopy that seems to be contributing to the patient’s dysphagia.

It is noteworthy that cases #2 and #3 were performed chronologically earlier than our highlighted case #1. While we did perform a transcervical approach, we did not remove hardware, performed cricopharyngeal myotomies, and covered the area of anastomosis from the nearby cervical hardware with only local tissue rotations rather than a muscle flap. Both of these patients have done well long-term and do not complain of dysphagia. In the majority of patients, this may be the approach indicated for management of ACSS-related diverticula. However, given our experiences with case #1 and the recent literature review, we advocate for a more aggressive approach with cervical hardware removal if possible, and rotation of an SCM flap. Less aggressive management of a traction diverticulum can result in persistent symptoms and postoperative complications such as recurrence. This can result in a more difficult case to manage. Therefore, a more aggressive and complete approach is likely to maximize success.

Limitations to this study include the retrospective nature, which restricts information gathering to what has previously been charted. Also due to the rare incidence of pharyngoesophageal diverticula formation following ACSS, there were a limited number of cases gathered at our institution during retrospective chart review. The authors hoped to augment our small number of cases with a thorough literature review to summarize and compare what has been previously reported.

## Conclusions

ACSS-related diverticula that form in patients with a history of prior anterior cervical spine surgery appear to be a type of traction diverticulum. Dense scar tissue adheres the pharyngeal or esophageal mucosa to the adjacent cervical spinal plate. Subsequent movement during swallowing results in a diverticulum forming. The mechanism of formation is different from Zenker’s diverticulum, and surgical management should be approached differently. Muscle flap coverage over the prior defect and/or cervical spine plate removal may be indicated for long-term resolution of patients’ symptoms.
